# Successful reconstruction of whole mitochondrial genomes from ancient Central America and Mexico

**DOI:** 10.1038/s41598-017-18356-0

**Published:** 2017-12-22

**Authors:** Ana Y. Morales-Arce, Courtney A. Hofman, Ana T. Duggan, Adam K. Benfer, M. Anne Katzenberg, Geoffrey McCafferty, Christina Warinner

**Affiliations:** 1 Department of Anthropology and Archaeology, University of Calgary, Calgary, Alberta T2N 1N4 Canada; 2 Department of Anthropology, University of Oklahoma, Norman, Oklahoma 73019 USA; 3 McMaster Ancient DNA Centre, Department of Anthropology, McMaster University, Hamilton, Ontario, L8S 4L8 Canada; 4 Department of Archaeogenetics, Max Planck Institute for the Science of Human History, Jena, 07743 Germany

## Abstract

The northern and southern peripheries of ancient Mesoamerica are poorly understood. There has been speculation over whether borderland cultures such as Greater Nicoya and Casas Grandes represent Mesoamerican outposts in the Isthmo-Colombian area and the Greater Southwest, respectively. Poor ancient DNA preservation in these regions challenged previous attempts to resolve these questions using conventional genetic techniques. We apply advanced in-solution mitogenome capture and high-throughput sequencing to fourteen dental samples obtained from the Greater Nicoya sites of Jícaro and La Cascabel in northwest Costa Rica (n = 9; A.D. 800–1250) and the Casas Grandes sites of Paquimé and Convento in northwest Mexico (n = 5; A.D. 1200–1450). Full mitogenome reconstruction was successful for three individuals from Jícaro and five individuals from Paquimé and Convento. The three Jícaro individuals belong to haplogroup B2d, a haplogroup found today only among Central American Chibchan-speakers. The five Paquimé and Convento individuals belong to haplogroups C1c1a, C1c5, B2f and B2a which, are found in contemporary populations in North America and Mesoamerica. We report the first successfully reconstructed ancient mitogenomes from Central America, and the first genetic evidence of ancestry affinity of the ancient inhabitants of Greater Nicoya and Casas Grandes with contemporary Isthmo-Columbian and Greater Southwest populations, respectively.

## Introduction

**Figure 1:**
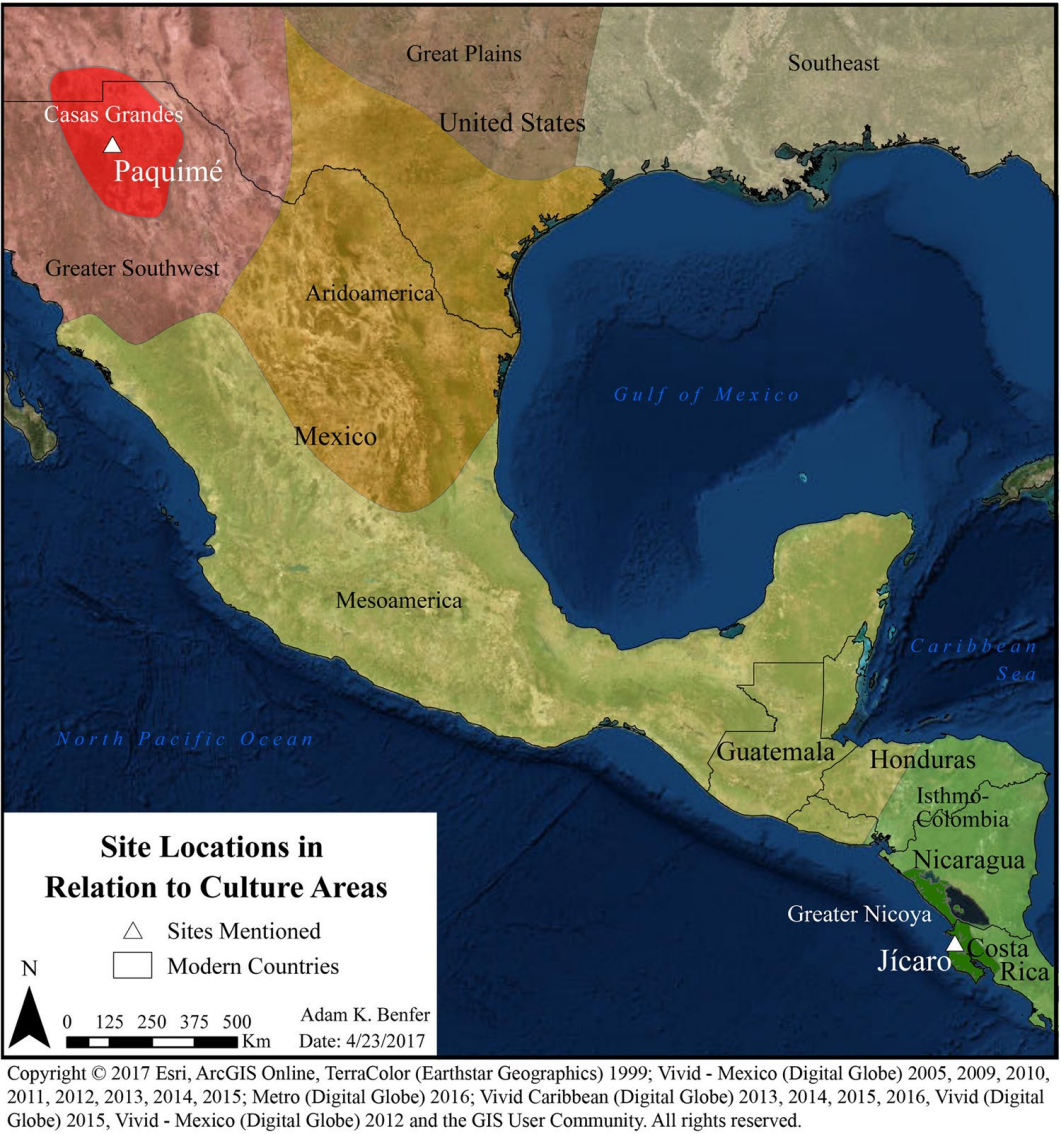
Archaeological sites location and corresponding culture areas as noted in the text. ArcGIS 10.4 software (http://www.esri.com/software/arcgis) was used to generate the figure. Service layer credits Esri, ArcGIS Online, TerraColor (Earthstar Geographics) 1999; Vivid - Mexico (Digital Globe) 2005, 2009, 2010, 2011, 2012, 2013, 2014, 2015; Metro (Digital Globe) 2016; Vivid Caribbean (Digital Globe) 2013, 2014, 2015, 2016, Vivid (Digital Globe) 2015, Vivid - Mexico (Digital Globe) 2012 and the GIS User Community.

The contemporary indigenous populations of Central America and Mexico present marked cultural, linguistic and genetic variation due to their diverse origins^1–5^. Today, nearly 300 different ethnic groups are found across this vast geographic region, which ranges from northern Mexico to southern Panama. This region can be further divided into four major cultural zones—the Greater Southwest, Aridoamerica, Mesoamerica, and the Isthmo-Colombian area—based on long-standing cultural, social, and linguistic differences^6–8^ (Fig. 1).

Archaeological evidence has shown continuous occupation of Central America and Mexico by humans since the end of the Pleistocene, providing a bridge that allowed the colonization of South America, and serving as an interethnic space for at least ten thousand years^1,5,9,10^. Following European contact in 1492, ethnohistoric records as well as linguistic and genetic evidence indicate population migration, decline, and the likely disappearance of several groups^11–18^. As a result, the pre-Contact distribution, movement, and autochthonous genetic diversity of populations within Central America and Mexico remain unclear^19,20^. This is particularly true for cultural borderland regions, such as those inhabited by the ancient Greater Nicoya and Casas Grandes cultures.

During the Classic (ca. A.D. 200–900) and Postclassic (ca. A.D. 900–1519) periods, complex Mesoamerican societies such as the Maya and the Triple Alliance (Aztecs) exerted considerable influence throughout Mesoamerica and beyond^21^. It has long been speculated that the borderland cultures of Greater Nicoya and Casas Grandes may represent Mesoamerican colonies or outposts^22–24^. Located at the far southern and northern peripheries of Mesoamerica, respectively, both cultures exhibit influences and evidence of long-distance exchange with Mesoamerican cores^25^, but current archaeological evidence is insufficient to identify the origins and migratory histories of these populations. The population histories of the Greater Nicoya and Casas Grandes cultural areas have been subject to intense debate, yet the archaeological record has failed to produce conclusive evidence to support or reject the different hypotheses.

Greater Nicoya is an ancient Central American cultural area known for its polychrome pottery with Mesoamerican influences during the Postclassic period, and it comprises the Isthmus of Rivas in Pacific Nicaragua and Northwestern Costa Rica (Fig. 1). At least by the Bagaces period (A.D. 300–800) Chibchan groups occupied this region, but beginning about A.D. 800, Mesoamerican influences are documented in the archaeological record^26^ through ceramic iconography and religious iconology^25,27^. At the time of European contact, early Spanish chroniclers documented speakers of Mesoamerican languages in Greater Nicoya; in the 1900s linguists confirmed that these languages belong to the Oto-Manguean and Nahua linguistic families of Chorotega-mangue, Subtiavas, and Nicaraos^28^. Based on these lines of evidence, archaeologists have argued that the main incursions of Oto-Manguean and Nahua-speaking Mesoamerican populations shared a common origin and dispersal by way of a migration from Central Mexico^23,25,27,29^. Unfortunately, there are no remaining speakers of these languages in southern Central America today^30^, and questions regarding this Mesoamerican migration are still debated^26,31^. The recovery of ancient mitochondrial genomes may clarify the genetic relationships of Postclassic Greater Nicoya inhabitants, including groups that are not longer extant.

The Casas Grandes (Paquimé) culture, located in the Northwest of Chihuahua, Mexico, first appeared as small settlements during the Viejo Period (A.D. 700–1200) and reached its apogee during the Medio Period (A.D. 1200–1450), but was abandoned by the mid-15^th^ century (A.D. 1450)^32–34^ (Fig. 1). The ancestry of Casas Grandes inhabitants remains unsolved^35^, but some have suggested that waves of Mesoamerican immigrants, possibly merchants, stimulated Paquimé’s development during the Medio Period. Evidence of human sacrifice, elaborate elite burials, and the ritual use of human bone have suggested influences from Mesoamerica^32,36^. Additionally, facilities for scarlet macaw (*Ara macao*) breeding at the site provides persuasive evidence for an enduring connection with Mesoamerican cultures because these birds are not found locally, but rather are native to the tropical lowlands of Mexico^32^. During the Medio Period, macaw feathers became important in the rituals of Southwestern cultures, and Paquimé played a central role in their production and distribution^37,38^. Finally, mathematical and astronomical models of construction, as well as I-shaped ball courts found at Paquimé are similar to those in Mesoamerica, but are infrequent farther north, suggesting a Mesoamerican influence at the site^32,39–41^.

It is uncertain whether the inhabitants from the Viejo Period and/or external influences from Mesoamerica or the American Southwest contributed to the flourishing of Paquimé^42^. On the one hand, archaeological evidence at the Convento site demonstrates material continuity from the Viejo to the Medio Period, reinforcing the idea of local development^43^. On the other hand, some have suggested that Paquimé shares a common cultural background with Greater Southwest groups such as the Mimbres culture^44–47^, and Paquimé’s mortuary practices, architecture, ceramics, and general symbolic and ceremonial behaviors are also common in the Greater Southwest^46^. However, estimations of biological affinity, based on discrete dental traits, have shown a weak relationship between Paquimé and Southwestern groups^48^. Genetic relationships inferred from ancient mitochondrial DNA sequences may provide valuable information on the population continuity and ancestry affiliation of the Casas Grandes culture.

Current understanding of the population history of Central America and Mexico is primarily based on modern mitochondrial DNA sequences from these regions. Previous studies have revealed the presence of four haplogroups—A, B, C, and D—whose distributions vary greatly between regions and different linguistic groups (Supplementary Table 1; Supplementary Figure 1). Today, Isthmo-Colombian populations have high frequencies of haplogroups A and B; haplogroup A, followed by haplogroup B, are the most common haplogroups in Mesoamerica, and Greater Southwest and Aridoamerican populations have high frequencies of haplogroup B, followed by haplogroup A. Haplogroups C and D are found as minor haplogroups in populations throughout these regions, except in the Isthmo-Colombian area, where they tend to be absent (Supplementary Table 1). This absence of C and D haplogroups in the upper Isthmo-Colombian area has not yet been confirmed for Pre-Columbian periods, and the time depth of this pattern is unknown.

In South America, recent ancient DNA (aDNA) studies have found a significant amount of diversity no longer traceable in living groups^49^, which suggest that this may also be the case for the rest of the Americas. Thus, the recovery of full mitochondrial genomes (mitogenomes) from ancient Central America and Mexico would greatly improve our understanding of the Pre-Columbian genetic structure and population history of this important region. Such information would be valuable for refining models of human colonization and expansion in the Americas, local processes of interaction and migration, and the impact of past pathogen outbreaks.

However, recovery of ancient human DNA is challenging within Central America and Mexico. The problem of poor preservation has been commonly reported for researchers trying to recover human aDNA from Mexico and the circum-Caribbean region^50–54^. The combined effects of temperature and humidity on DNA preservation assume particular importance in tropical regions^55–57^, and the likelihood of recovering aDNA decreases as one approaches the equator^58^ because hot climates accelerate the rate of DNA degradation^59^. Thus, the recovery of authentic ancient molecules is highly challenging in Central America and Mexico.

In recent years, the availability of new high-throughput sequencing (HTS) technologies, together with improved DNA polymerases and capture-enrichment methods, have allowed the recovery of DNA molecules from highly degraded material and could thus offer a solution for recovering aDNA from tropical environments^60,61^. However, to date these methods have not been applied to ancient human remains in Central America, and they have only been applied to a limited extent in Mexico^49^.

**Figure 2:**
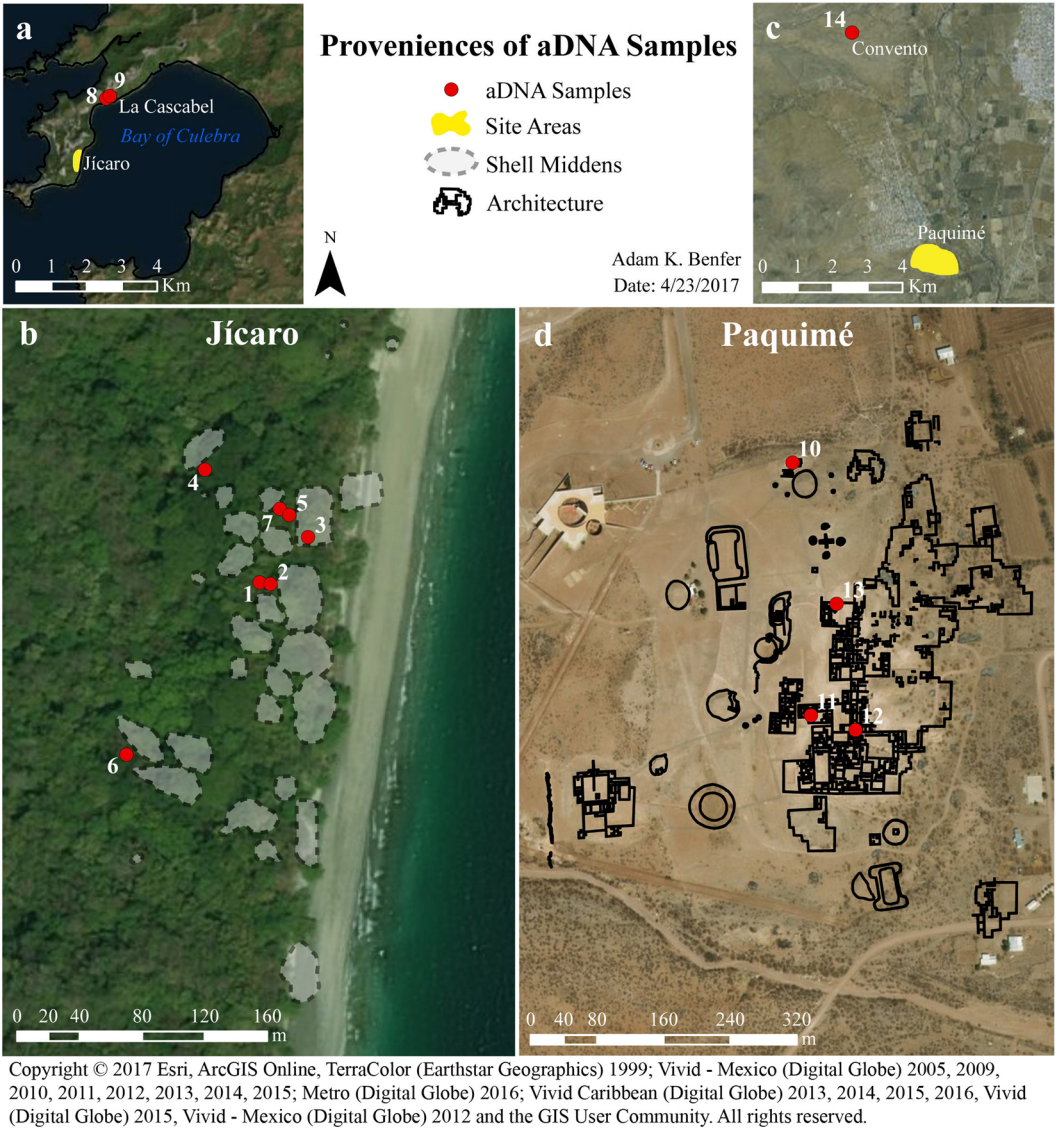
Location of the archaeological sites and aDNA samples for each excavated area. (**a**) Locations of the sites Jícaro, La Cascabel, and La Cascabel aDNA samples, (**b**) aDNA samples distribution excavated from Jícaro, (**c**) Location of Convento site and Paquimé, (**d**) aDNA samples distribution excavated from Paquimé. ArcGIS 10.4 software (http://www.esri.com/software/arcgis) was used to generate the figure. Service layer credits Esri, ArcGIS Online, TerraColor (Earthstar Geographics) 1999; Vivid - Mexico (Digital Globe) 2005, 2009, 2010, 2011, 2012, 2013, 2014, 2015; Metro (Digital Globe) 2016; Vivid Caribbean (Digital Globe) 2013, 2014, 2015, 2016, Vivid (Digital Globe) 2015, Vivid - Mexico (Digital Globe) 2012 and the GIS User Community.

In this study, we explore the potential of these techniques for the recovery of aDNA from fourteen individuals at four archaeological sites in Central America and Mexico: Jícaro and La Cascabel, Greater Nicoya, in northwest Costa Rica (A.D. 800–1250) and the sites of Paquimé and Convento, Casas Grandes, in northwest Mexico (A.D. 700–1450) (Table 1; Fig. 2). The Greater Nicoya sites are located in non-temperate regions and the two sites from Casas Grandes are located in an arid region, thus offer the opportunity to test the effectiveness of using advanced molecular techniques to recover DNA from low latitudes and warm climatic conditions. We demonstrate that sequence capture-enrichment coupled with HTS enables the successful recovery of full mitogenomes from Central American and Mexican skeletal material, including samples for which conventional PCR-based techniques were unsuccessful. Additionally, we show that the Costa Rican samples exhibit a very high rate of DNA damage, which is consistent with the tropical climate of southern Central America.

**Table 1:** Archaeological sites dates and number of samples processed.

Site	Period	Dates	N	Sample IDs
*Greater Nicoya*				
Jícaro	Sapoá Period	A.D. 800–1250	7	1–7
La Cascabel	Sapoá Period	A.D. 800–1250	2	8–9
*Casas Grandes*				
Convento	Viejo	A.D. 700–1200	1	14
Paquimé	Medio	A.D. 1200–1450	4	10–13
***Total***			***14***

## Results

### DNA sequencing results

Attempts to obtain sequences by conventional PCR amplification and Sanger sequencing yielded poor results. Of the 6 samples tested, only two samples (Sample IDs_9 and 10) yielded DNA fragments whose variants corresponded to those expected in Native American mitochondrial haplogroups. Additionally, replicate amplification was possible only for Sample ID_10, which successfully amplified four times for each of the fragments (4x) and showed variants characteristics of the haplogroup C (Table 2; GenBank accession number KY400154). Two samples did not amplify any of the overlapping fragments (Samples IDs_6 and 7^c^), and the remaining two samples (IDs_2 and 8) amplified fragments that did not include any of the Native American haplogroup variants, with inconsistent results in each attempt to verify amplifications (ID_8), suggesting contamination (Table 2).

**Table 2:** Laboratory results PCR vs. in-solution capture.

Sample ID	Site	Individual	Extraction 1-Calgary	Extraction 2-LMAMR	PCR amplification^a^ (X)	#Range (bp) covered	Mitogenome in-solution capture (HTS)*,^b^	Haplogroup	
								PCR	HTS
1	Jícaro	31–127		LM3			**Full coverage**		B2d
2	Jícaro	1–133	LM3		Contamination (1x)	16267–16410 (143 bp)	**Full coverage**		B2d
3	Jícaro	64		RM2			**Full coverage**		B2d
4	Jícaro	22–33		RM3			Insufficient coverage		
5	Jícaro	24–96		LPM2			Insufficient coverage		
6	Jícaro	39–154	LI2	RI1	Negative		Insufficient coverage		
7^**c**^	Jícaro	24–130	RM1		Negative		Insufficient coverage		
7^**d**^				RPM1	Negative		Insufficient coverage		
8	La Cascabel	3–4	RPM1	RM1	Contamination (3x)	16112–16410 (298 bp)	Insufficient coverage		
9	La Cascabel	58–26	RM3	RPM2	**Positive** (**1x**)	16112–16410 (298 bp)	Insufficient coverage	C	
10	Paquimé	14–1 A	LI2		**Positive** (**4x**)	**15989–16410** (**421 bp**)	**Full coverage**	C	C1c5
11	Paquimé	20–13		RI1			**Full coverage**		C1c1a
12	Paquimé	2–16		LI1			**Full coverage**		B2f
13	Paquimé	25–6		LI2			**Full coverage**		C1c1a
14	Convento	16		LI2			**Full coverage**		B2a

Notes: ^a^PCR was performed on DNA obtained from Extraction 1. ^b^With the exceptions of samples 2, 7^c^, and 10, HTS was performed on DNA obtained from Extraction 2. ^c^Sample duplicate 7^c^ = RM1, Extraction 1. ^d^Sample duplicate 7^d^ = RPM1, Extraction 2. *See Supplementary Table 3.

By contrast, using HTS techniques recovery of whole mitogenomes was successful to a sequence coverage depth of >8x for one third of the Greater Nicoya samples (n = 3); the remaining samples yielded fewer sequences and insufficient coverage for further analysis (Supplementary Table 3). In the case of sample ID_2, DNA sequence-capture and HTS yielded a complete mitogenome with 12-fold coverage and a low estimated contamination rate (4%) from an extract that had previously failed to yield reliable sequences using PCR (Table 2). Here, DNA-sequence capture appears to have overcome contamination that otherwise made sequences generated by PCR unusable (Table 3). For sample 7, two different DNA extracts were generated using extraction methods 1 and 2, but neither PCR nor DNA sequence-capture was successful for this individual (Table 2). Finally, although the individuals from La Cascabel did not produce sufficient data for mitogenome reconstruction, it should be noted that PCR amplification of sample ID_9 yielded 287 bp of the HVR-I in three overlapping amplicons. The consensus sequence contained the variants 16223T, 16325C, 16298C, and 16327T, which are consistent with haplogroup C. However, the fragment from HVR-II that confirms this haplogroup with a deletion in 290–291 did not amplify, and the results could not be replicated; thus, the authenticity of this result cannot be confirmed.

**Table 3:** Mitochondrial reads in the libraries, average fragment length, coverage, and contamination estimate, for the samples in which HTS sequencing was successful. (See Supplementary Table 3 for extended version).

Site	Sample ID	Raw reads	Reads frag. length min 35 bp	Mapped unique reads (min35 MQ30)	Average frag. length	% on target*	Mean coverage depth	Contamination estimate
Jícaro	1	3630538	3474724	5652	65	0.16	24.9	0.06
	2	2123101	2061034	3225	56	0.15	12.0	0.04
	3	1881431	1750773	19384	60	1.10	77.8	0.04
Paquimé	10	2118959	2092514	1671	84	0.08	8.7	0.04
	11	712746	695257	94031	80	13.52	495.4	0.04
	12	2297010	2250578	267126	85	11.87	1459.5	0.01
	13	1601441	1499544	25108	64	1.67	110.5	0.05
Convento	14	140292	137474	3659	83	2.66	19.3	0.04

*This percentage was estimated as the number of mapped reads with a minimum fragment length of 35 bp (mapped quality 30) divided by the total of raw reads.

For the sites of Convento and Paquimé, whole mitogenomes were successfully obtained for all five tested samples (Table 2). In one case (sample ID_10), DNA extracted at the University of Calgary that was successfully amplified using overlapping primer sets also yielded a full mitogenome at the University of Oklahoma. Both methods resulted in sequences consistent with haplogroup C, but the full mitogenome reconstruction allowed much greater resolution of the haplogroup (Table 2). The other samples from Paquimé were not analyzed using targeted PCR.

### Mitogenome analysis

For the eight successfully reconstructed mitogenomes, the mean depth of coverage was 38.0x (12.0–77.8x) for the three Jícaro samples and 418.6x (8.7–1459.5x) for the five Casas Grandes samples (Table 3). The percentage of on-target reads was lower in Jícaro (0.15–1.10%) than in Paquimé (0.08–13.52%) (Table 3). The eight mitogenomes were assigned to eight distinct haplotypes within the haplogroup families B and C and fall within the expected mitochondrial diversity of Central America and Mexico. Interestingly, no individuals belonged to haplogroup A, the most common haplogroup found in Mesoamerican populations today.

The three Jícaro individuals all belong to haplogroup B2d; however, each has a distinct sequence and thus the three individuals are not close maternal relatives. A deletion defines this haplogroup at position 498 of the revised Cambridge reference sequence (rCRS) and it is estimated to have emerged ~2,900 years ago^62^. To date, haplogroup B2d has been reported in the Arawak group of the Wayuu in Colombia and the Chibchan Ngöbe group in Panama, but not in Mesoamerican groups^63,64^.

The five mitogenomes obtained from the Casas Grandes sites belonged to four haplogroups B2a, B2f, C1c1a, and C1c5. Although two individuals shared the haplogroup C1c1a, they have distinct sequences (Supplementary Table 4), and are thus not close maternal relatives. The older sample, from the Convento site, presented the halogroup B2a, which is estimated to have emerged ~12,100 years ago^62^, and it has been observed in Native American groups such as the Tsimshian, Chippewa, and Pima people, but it has not yet been reported in Mesoamericans. The only haplogroup B observed in Paquimé corresponded to the B2f, which has been reported for Mexican-American individuals but not yet for populations resident in Mesoamerica^62,65–68^. Similarly, the haplogroup C1c1a observed in Paquimé has been previously described for U.S. Native American groups and for the Pima Bajo people in Mexico, but not for any Mesoamerican groups^66,69,70^. The haplogroup C1c1 has been estimated to have emerged by ~7,300 years ago^62^. Finally, the last haplogroup observed at Paquimé, C1c5, is also found in Mexican Americans, and similarly, this has not yet been reported for Mesoamerican groups^68,71^. Overall, the availability of whole mitogenome sequence information for contemporary populations is very limited in the Americas, and a larger mitogenome database of modern groups would be necessary for more reliable comparisons.

### Authenticity of Ancient Human DNA

For each mitogenome, the mean fragment lengths of DNA were short, 56–85 bp, which is typical of ancient DNA^59,72^. The average fragment length of the Jícaro samples ranged from 56–65 bp, while the Casas Grandes samples were slightly longer, ranging from 64–85 bp (Table 3; Supplementary Figure 2).

**Figure 3:**
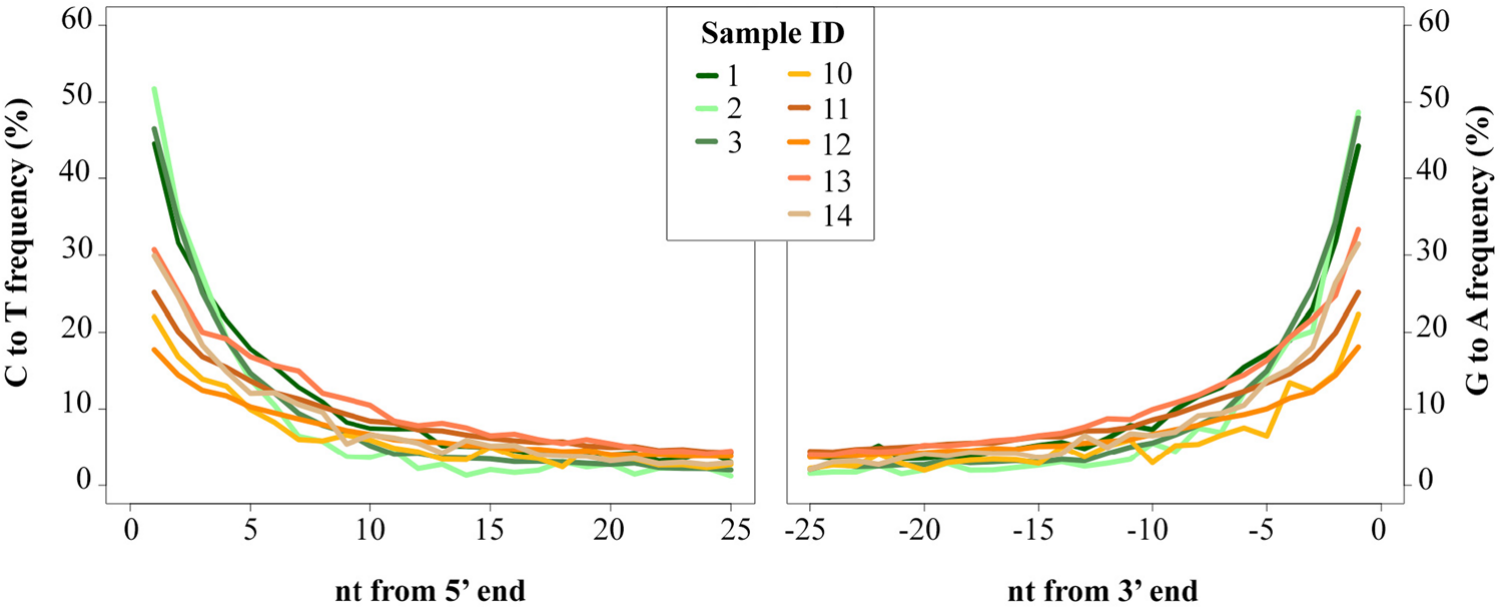
Terminal deamination (C → T) patterns observed in human mitochondrial sequences recovered from Greater Nicoya (green) and Casas Grandes (orange) archaeological sites in this study. Note that terminal deamination rates are much higher in the Greater Nicoya samples compared to the Casas Grandes samples, a pattern that is consistent with the lower latitude and higher mean annual temperature of Greater Nicoya site of Jícaro, Costa Rica compared to the Casas Grandes sites of Convento and Paquimé in northwestern Mexico.

The authenticity of human reads obtained from the archaeological dental samples was further evaluated by examining damage patterns typical of ancient DNA^73,74^. All eight mitogenomes exhibited reads with a high rate of terminal C → T transitions consistent with cytosine deamination (Fig. 3), and an overrepresentation of purines at the −1 position consistent with depurination followed by strand nicking (Supplementary Figure 3). The terminal deamination rates observed in the Paquimé samples range from 17–30%, while the terminal deamination rates for the Jícaro samples exceeded 40%, ranging from 44.5–51.7%. Such extremely high terminal C → T transition rates have only previously been described for archaic hominin mitogenomes^73^ (Fig. 3).

Modern contamination was estimated using the program Schmutzi^75^. For all samples, contamination estimates were low, ranging from 1–6% (Table 3). At these levels, contamination is unlikely to affect the determination of consensus sequences for high coverage genomes. Finally, all lab analysts involved in the study were tested and shown to have Eurasian haplogroups inconsistent with the haplogroups identified in this study.

## Discussion

Ancient mitochondrial genomes are difficult to obtain from southern Central America, as noted by the low success rates of samples from the archaeological sites of Jícaro (30%) and La Cascabel (0%). However, it can be even more challenging if applying PCR methods, which in the case of the Costa Rican samples did not yield successful results when targeting overlapping fragments of the HVR-I using PCR amplification. These results are consistent with previous studies that predicted poor DNA preservation and retrieval from tropical areas^58,76,77^. Most previous human ancient DNA studies in Central America and Mexico have applied PCR, cloning, and Sanger sequencing methods, with mixed results^50,51,78–85^. These methods are limited in their ability to estimate DNA damage precisely, and contamination is difficult to exclude^86,87^. In this study, by applying in solution capture enrichment techniques and HTS methods, we successfully recovered authentic DNA sequences from ancient samples at three different latitude sites, including Paquimé and Convento where all tested samples yielded complete mitogenomes. Therefore, we demonstrate that by applying HTS techniques, it is possible to recover and authenticate ancient mitogenomes from Central America and Mexico through the quantification of DNA damage and contamination levels.

There were considerable differences in the levels of damage observed in mitochondrial DNA molecules between Jícaro and the Casas Grandes sites of Paquimé and Convento. For example, the terminal C → T deamination rate was higher in the Jícaro samples than in the Paquimé samples (Fig. 3), and the mean DNA fragment lengths were shorter for the Jícaro samples as well (Supplementary Figure 2a). These patterns correspond with DNA damage predictions based on climatic conditions^59^. The sites of Jícaro and La Cascabel are located in a region highly affected by the main factors that accelerate the rate of hydrolytic depurination, namely the presence of chemically available water and warm temperatures^57,88–90^. In contrast, the higher altitude, latitude, and drier climatic conditions of the Paquimé and Convento sites may have contributed to the better molecular preservation of the Casas Grandes samples. It is also noteworthy that the Convento and Paquimé samples showed a higher frequency of fragments larger than 100 bp in length compared to the Jícaro samples (Supplementary Figure 2b). Longer fragment lengths could explain why PCR was able to amplify overlapping fragments (4x) of the HVR-I from the Paquimé sample ID_10 (Table 2). Given the highly degraded nature of the DNA recovered from the Greater Nicoya sites, further methodological optimization of capture incubation temperatures^91^ and the use of single-stranded library preparations^60^ may be needed to improve the efficiency of mitogenome recovery from this region.

Additional analysis to evaluate the conditions promoting preservation in the burials are recommended, and variance in DNA recovery and coverage between samples should be interpreted with burial context information. For example, the skeletal material for the sample ID_10 was not found in a burial but rather was very fragmented and scattered on the ground, with evidence of burning^32,92^. This exposure may have affected overall DNA recovery, including mitogenome coverage and percentage on target—the lowest (8.7x and 0.08%, respectively) among the Casas Grandes samples. Another sample, ID_14, yielded the second-lowest coverage for Casas Grandes and a low percentage on target (19.3x and 2.66%, respectively). This is from the Convento site, which is generally earlier than the burials at Paquimé^93^, and thus this factor could explain its lower coverage and percentage on target results compared to the more recent Paquimé samples. The haplogroup for this individual, B2a, contains a polymorphism at position 16483 A that is specific to the Americas and which has been found in most of the Southwest populations and the northwestern Tsimshian (British Columbia)^65,94^. This haplogroup may have originated ~12,100 years ago in North America, and today it is present at high frequency in Southern Athapaskan groups (Apache: 14.8%; Navajo: 15.1%)^65^. Interestingly, the distinctive 16483 A polymorphism of haplogroup B2a has not been found in Mesoamerican or Central American groups^94,95^. Overall, haplogroup C was observed in three out of five Paquimé samples, and this corresponds to previous observations in ancient populations. For example, haplogroup C is more frequent in the Greater Southwest and Aridoamerica than in Mesoamerica, as reported by aDNA studies for the ancient Ancestral Puebloan populations such as those of Fremont, Basketmakers, the Tommy Site, and Mimbres^20,96–98^. Further sequencing from ancient Central American and Mexican areas would be necessary so as to clarify the distribution of this haplogroup and others.

The mitogenomes successfully obtained from Jícaro, Greater Nicoya, suggest that HTS methods could provide a productive approach for further investigating population history in Central America. Haplogroup B2d, which was assigned to all three of the Jícaro mitogenomes, is a clade rooted in haplogroup B2, the second most frequent haplogroup after haplogroup A2 in contemporary Central American and Mesoamerican populations. Haplogroup B2 ranges in frequency between 4% in Chibchan speakers, such as Huetar from Costa Rica, to 29% in the Panamanian Kuna, and up to 92% among the Rama on the Caribbean side of Nicaragua^99–101^. Similarly, Mesoamerican populations have haplogroup B2 in frequencies ranging from 10% up to 52% in the Nahua and the Maya groups, and it is also represented in the Otomi Valle and the Tepehua people^102,103^. However, although haplogroup B2 is widely distributed, the subgroup B2d that was identified in the Jícaro samples appears to have a more limited distribution in lower Central America. Caution should be exercised when interpreting these results, however, because most mitochondrial data for Central American indigenous groups corresponds to control region markers only and not entire mitogenomes, and thus haplogroup assignment below the level of B2 is not possible given currently available data from many of these populations. Nevertheless, for contemporary populations where further haplotyping information is available, the subgroup B2d has only been reported in the Arawak group of the Wayuu in Colombia and the Chibchan Ngöbe group in Panama^63,64^.

DNA sequence-capture and HTS allow major gains in mtDNA recovery from ancient Central American and Mexican skeletal remains, as compared to conventional PCR-based approaches. In this study, we applied these techniques to fifteen ancient Central American and Mexican skeletal samples and successfully reconstructed full mitogenomes for eight individuals. The DNA obtained from the Central American samples was particularly degraded, with very short fragment lengths and high rates of terminal miscoding lesions resembling samples from temperate regions that are thousands of years older. Future studies aiming to recover additional mitogenomes or nuclear DNA from ancient Central American sites are advised to take this high degree of damage into account during the study design.

Full mitogenome sequences provide a level of maternal ancestry resolution that far exceeds what is possible to achieve from haplogroup typing studies based on individual coding region polymorphisms or HVR sequences alone, and it is this level of genetic resolution that is necessary to unravel the complex population history of Central America and Mexico. Here we demonstrate that whole mitogenome reconstruction is possible from ancient human remains in this region, despite the challenges of preservation. The results of this study provides the first genetic evidence of the ancestry affiliations of the ancient borderlands cultures of Greater Nicoya and Casas Grandes. The mitochondrial haplogroups observed within these ancient groups tentatively suggest a greater degree of maternal genetic affinity with contemporary Isthmo-Columbian area and Greater Southwest populations, respectively, than with contemporary Mesoamerican populations, but additional high resolution mitochondrial sequence data from contemporary indigenous American populations are necessary to resolve these relationships with greater confidence. Finally, this study presents a powerful and repeatable methodology for obtaining human aDNA in Central America and tropical regions, which may be used to reveal the ancestry, diversity, and movements of ancient Mesoamerican and Central American cultures through time.

## Materials and Methods

### Archaeological sites background

The four archaeological sites analyzed in this study date to the period A.D. 700–1450 (Table 1). The two Greater Nicoya sites in northwest Costa Rica, Jícaro, and La Cascabel (Fig. 2a), correspond to the Sapoá Period (A.D. 800–1250)^104^. Excavations at Jícaro were conducted over three seasons between 2005 and 2008^105^ (Fig. 2b) while excavations in La Cascabel were conducted in 2007^106^.

Samples analyzed from the northern Mexican Casas Grandes site of Paquimé date to the Medio Period (AD 1200–1450), and one sample from the Convento site dates to the Viejo Period (A.D. 700–1200). Paquimé and Convento are located in the cultural borderlands between the American Southwest (Greater Southwest and Aridoamerica) and Mesoamerica and were systematically excavated from 1958 to 1961^32^ (Fig. 2c,d).

Genetic investigation of skeletal material from the sites of Jícaro, La Cascabel, Paquimé, and Convento, additionally present an opportunity to observe the impact of climate on ancient DNA preservation in northern Mexico and Central America. Jícaro and La Cascabel are found in the tropical dry forest of northwest Costa Rica, 10.6° latitude from the Equator, with an elevation of 70 meters above sea level (masl) and annual temperatures 26–33 °C. The average annual precipitation for this area is >1500 mm^107^. In contrast, Paquimé and Convento are found in the dry climate of Chihuahua, at latitude of 30.3° and elevation of 1457 masl, with annual temperatures between −10 °C and 42 °C. The average annual precipitation for this area is approximately 326 mm^108^. Given the differences between these two sites, DNA preservation is expected to be poorer at the Costa Rican sites, where higher humidity, lower altitude, and equatorial latitude are likely to accelerate DNA decay.

### Archaeological samples

A total of fourteen individuals were selected for this study (Table 1), with one tooth analyzed per individual, except for Jícaro sample ID_7 for which two teeth were tested (Table 2). Thus, fifteen teeth were processed in total. Nine individuals from Greater Nicoya were sampled at the National Museum of Costa Rica. The sites of Jícaro and La Cascabel were selected because of their notable skeletal preservation^105,106^, which included the preservation of aging indicators on the pelvic auricular surface and dental occlusal surfaces^109^. The five Casas Grandes individuals were sampled from Museo de las Culturas del Norte, INAH Chihuahua, as part of a larger project on Casas Grandes bioarchaeological research at the University of Calgary. A map with the approximate locations from where the samples were excavated (Fig. 2) was produced using Esri’s ArcMap^TM^ 10. The site maps were redrawn and overlaid on the satellite imaginary basemap provided by Esri.

Permission to sample the Casas Grandes material was provided by the INAH-Chihuahua and Museo de las Culturas del Norte (Paquimé), and ethical review for genetic analysis was performed and approved by the University of Calgary Conjoint Faculties Research EthicsBoard (REB15-2680). For the Costa Rican material, permission for sampling and analysis was granted by the National Museum and Minister of Culture of Costa Rica.

### DNA extraction

Archaeological dental samples were processed in dedicated aDNA facilities at the University of Calgary’s Ancient DNA Laboratory and at the University of Oklahoma’s Laboratories of Molecular Anthropology and Microbiome Research (LMAMR). At both facilities, all sample decontamination steps and DNA extraction methods followed established procedures to prevent contamination and specific details are provided below. A non-template extraction control (negative control) was processed alongside the experimental samples during all analytical steps to monitor for the presence of contamination.

#### Ancient DNA Laboratory, University of Calgary

Prior to beginning this study, 26 samples from Jícaro and La Cascabel and one sample from Paquimé were screened for DNA preservation. Only six of these samples (Sample IDs_2, 6, 7, 8, 9, and 10 in Table 2) were determined to be suitable for further study. DNA extraction was performed as follows. Samples were submerged in 6% sodium hypochlorite solution for 10 minutes, rinsed twice with ultrapure water, and irradiated with 254-nm ultraviolet light for 30 min per side to reduce surface contamination. Afterward, whole samples were pulverized, without removing the crown, and a total of 1–2 g of tooth powder was transferred to a 15 ml tube with 5 ml of extraction solution (0.5 M EDTA pH8, 0.12% SDS, 0.5 mg/ml proteinase K) and incubated at 50 °C overnight with agitation. Following this, DNA was purified and concentrated using a MinElute silica spin column protocol^110^ and eluted twice into 50 μl aliquots of TET buffer.

#### LMAMR, University of Oklahoma

DNA was extracted from twelve samples in a dedicated aDNA laboratory at the University of Oklahoma, including four samples that were previously extracted at the University of Calgary (Table 2). Prior to extraction, dental calculus was removed from all dental samples, and the tooth surface was gently cleaned with a 2% sodium hypochlorite solution followed by ultrapure water. The tooth root surface was removed by mechanical abrasion using a Dremel rotary tool, separated from the crown, UV-irradiated on each side for 1 min, and then pulverized to a coarse powder. Approximately 100 mg of tooth powder was pre-digested in a 0.5 M EDTA solution for 15 min and decanted to further remove contaminants. The cleaned tooth powder was then incubated in a solution of 1 ml of 0.5 M EDTA for 24 h at room temperature under agitation, followed by the addition of 100 µl of Proteinase K (Qiagen) and further incubation until digestion and decalcification were complete. DNA was isolated following a silica adsorption protocol^111^ using a Qiagen MinElute PCR Purification kit and twice eluted into 30 µl of EB buffer (10 mM Tris-Cl, pH 8.5) for a combined elution volume of 60 µl.

### PCR amplification of the mitochondrial hypervariable region

At the University of Calgary, amplification of approximately 600 bp of the mitochondrial hypervariable regions I and II (HVR-I and HVR-II) was attempted for 6 samples (Table 2) extracted there. The region was amplified by targeted PCR in four overlapping segments of approximately 140 bp each, with the following primer pairs: F15989 - R16158, F16112 - R16251, F16190 - R16322, F16268 - R16410. Primer design followed Gabriel and colleagues (2001) except for R16251 [5′-GGA GTT GCA GTT GAT GT-3′]. The PCR reaction final volume was 30 µl and contained 2.5 mM MgCl_2_, 0.2 mM dNTP, 1.0 mg/ml Bovine Serum Albumin (BSA), 0.3 µM forward and reverse primer, and 2.5 U AmpliTaq Gold^TM^ enzyme. A total of 3–5 µl of DNA extract was added to each PCR reaction, which was amplified according to the following conditions: initial denaturing at 95 °C for 12 min, followed by 40 cycles at 95 °C for 30 sec, 50 °C for 30 sec, 72 °C for 30 sec, followed by a final extension of 72 °C. Electrophoresis on 2% agarose gels was used to visualize positive amplifications of targeted fragments. PCR products were sequenced using forward and/or reverse primers at Eurofins Genomics (Louisville, KY). The resulting chromatograms were edited using ChromasPro 2.6.2 software to remove primer sequences and low quality bases and aligned to the revised Cambridge reference sequence (rCRS NC_012920; Andrews *et al*.^112^ using BioEdit 7.2.5 software to determine variant positions.

### HTS library construction

Illumina sequencing libraries were prepared for a total of fifteen samples (Table 2). All initial library preparation steps were performed in the University of Oklahoma’s LMAMR dedicated ancient DNA facility following previously published protocols^113^, with minor modifications. In brief, up to 30 μl of sample extract (to a maximum of 100 ng input DNA) (Supplementary Table 2) was constructed into Illumina libraries using the NEBNext DNA Library Prep Master set for 454 (E6070; New England Biolabs). Library preparation was modified from the manufacturers’ instructions, decreasing the total volume to 50 µl, and also replacing SPRI bead purification with silica column purification (Qiagen MinElute PCR Purification kit). Blunt end adapters (IS1/IS3 and IS3/IS2) were prepared and used for ligation at a final concentration of 0.6 µM in a final volume of 50 µl. The Bst polymerase fill-in reaction was inactivated by heating at 80 °C for 20 min, followed by freezing overnight.

For each archaeological sample, the library was amplified in triplicate using 0.75 µl of each indexed primer at 10 µM, 12.5 µl of KAPA HiFi Uracil+ (Kapa Biosystems), 1 µl of BSA at 2.5 mg/ml, 4 µl of library template, and 6 µl of water for a final volume of 25 µl. PCR conditions were as follows: initial denaturation at 95 °C for 5 min, followed by 10–16 cycles of denaturation at 98 °C for 20 s, annealing at 60**°**C for 15 s, and elongation at 72**°**C for 30 s, followed by a final elongation at 72**°**C for 1 min. The number of cycles necessary for the library amplification was determined by Real-Time PCR, selecting the midpoint of the exponential phase of amplification for each sample. All negative extraction controls were amplified for 16 cycles, equal to the greatest cycle number for an archaeological sample. The success of library preparation was determined by visualization on a 2% agarose gel and the triplicate reactions were pooled. The reactions were purified using the AMPure substitute protocol^114^ and eluted into 10 ul RNase free water.

### Mitogenome in-solution capture and sequencing

Mitochondrial in-solution capture was performed on all shotgun libraries following the protocol described by MYbaits V3.0, with minor modifications. Briefly, each pool of libraries was incubated with RNA probes and buffers for 48 h in a touchdown enrichment that started at 65 °C for 16 h, then 60 °C for the next 16 h, and finally 55 °C for the last 16 h. Libraries and beads were incubated for 30 min at 55 °C and Wash Buffer 2.2 was heated to 55 °C. The subsequent steps of beads washing and releasing from the RNA bait were conducted as described in the MYbaits V3.0 protocol. Real-Time PCR was used to determine the cycles necessary for post-capture amplification, and post-capture PCR was performed in triplicate reactions of 25 µl each containing the following: 1X KAPA HiFi Uracil+, 0.1 mg/ml BSA, 0.3 µM of each Primer-Illumina ext, 4 µl of template, and water. PCR conditions were as follows: initial denaturation at 98 °C for 2 min, followed by 14–25 cycles of denaturation at 98 °C for 20 s, annealing at 60**°**C for 30 s, and elongation at 72 **°**C for 30 s, followed by a final elongation at 72**°**C for 5 min.

The amplified post-capture libraries were quantified using a Bioanalyzer 2100 DNA 1000 assay (Agilent) and pooled into equimolar ratios. Size selection to remove primer dimers was performed using a PippinPrep (Sage Science), and the combined pool was sequenced using Illumina HiSeq v2 2 × 100 bp chemistry at the Yale Center for Genome Analysis (YCGA).

### HTS data analysis

Sequence reads were processed using a bioinformatics pipeline optimized for ancient DNA analysis. Post-capture paired reads were merged and adapters were trimmed with the program leeHom using the ancient DNA flag^115^. The reads were mapped to the rCRS^112^ using a modified version Burrows Wheeler Aligner (-n 0.01, -o 2, -l 16500)^116^ (https://github.com/mpieva/network-aware-bwa). Reads were filtered to those that were mapped and merged or properly paired (https://github.com/grenaud/libbam) and duplicate molecules collapsed on the basis of identical 5′ and 3′ mapping positions (https://bitbucket.org/ustenzel/biohazard). Mapped reads were further filtered to those with a minimum length of 35 bp and minimum quality of 30^117^. Schmutzi^75^ was utilized to estimate endogenous mitochondrial consensus sequences and contamination (−qual 10) using the comparative Eurasian database. Automated indel calls were manually confirmed directly from the read pileups and corrected to avoid alignment errors. The assignment of mitochondrial haplogroups was performed using Haplogrep 2.0^118–120^. Ancient DNA damage patterns were assessed using MapDamage v.2^74^.

### Data availability

Genetic data are available in the NCBI Short Read Archive (SRA) under the BioProject accession PRJNA360145 (SRA accessions SAMN06204047 to SAMN06204061).

## Electronic supplementary material

Supplementary information

Supplementary Table 3
