# Supplementary material for: Successful reconstruction of whole mitochondrial genomes from ancient Central America and Mexico
Source: Sci Rep. 2017 Dec 22;7:18100. doi: 10.1038/s41598-017-18356-0 (PMC5741722; doi:10.1038/s41598-017-18356-0)
Supplement: Supplementary file 1 — Supplementary information [file 41598_2017_18356_MOESM1_ESM.pdf]

Supplementary Information

Successful reconstruction of whole mitochondrial genomes from ancient Central America and Mexico

Ana Y. Morales-Arce*, Courtney A. Hofman, Ana Duggan, Adam K. Benfer, M. Anne Katzenberg, Geoffrey McCafferty, Christina Warinner*

*SI correspondence to:
Ana Y. Morales-Arce, aymorale@ucalgary.ca
Christina Warinner, warinner@shh.mpg.de

Supplementary Figure 1. Mitochondrial haplogroup frequencies of selected indigenous American populations. References are provided in Supplementary Table 1. Map modified from Pixabay (https://pixabay.com/en/world-map-continent-country-117174/)

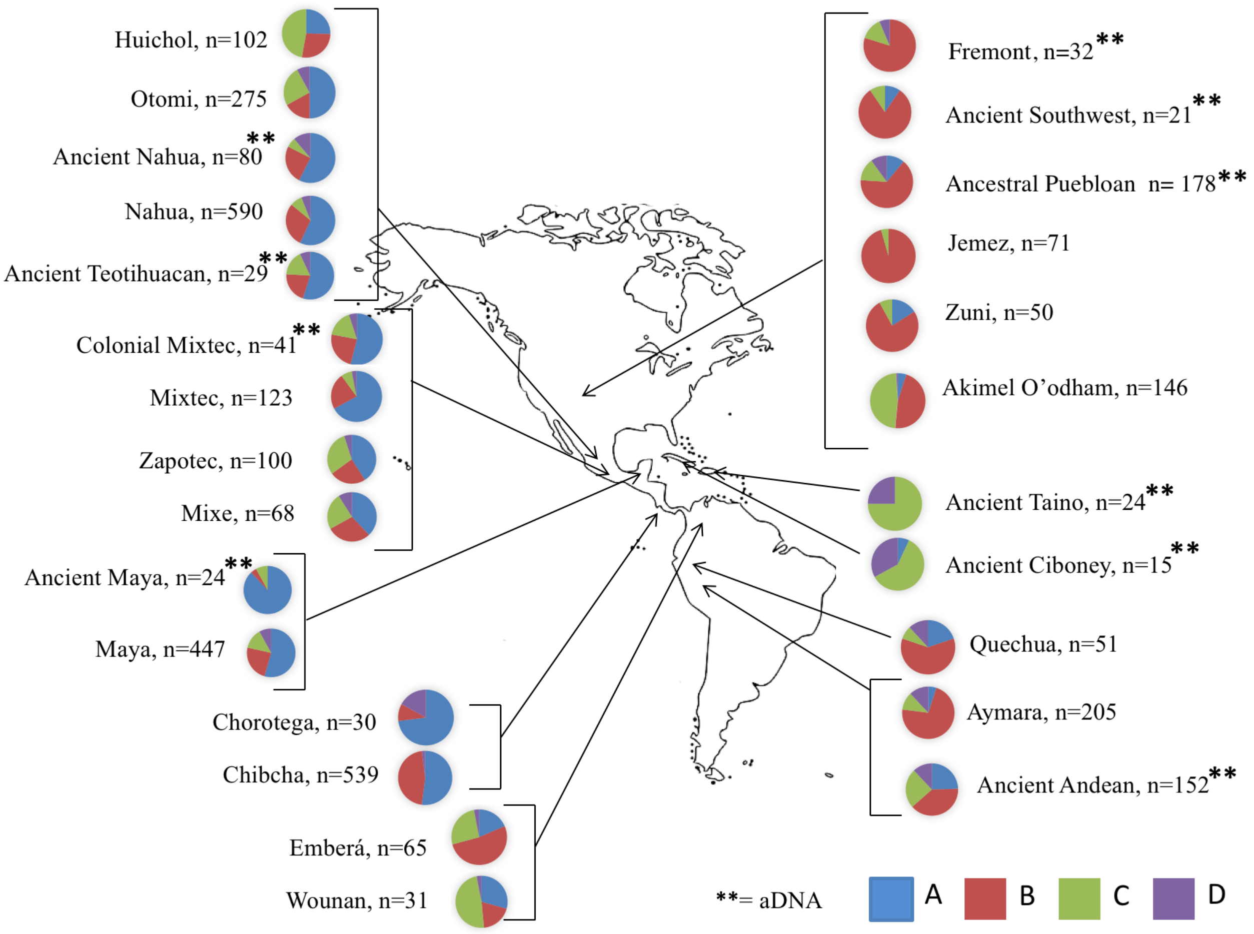

Supplementary Figure 2. Number of reads and sequence length distribution of unique mitochondrial DNA sequences from a) Jícaro, and b) Paquimé and Convento. Note that only merged reads were used to produce this figure.

a

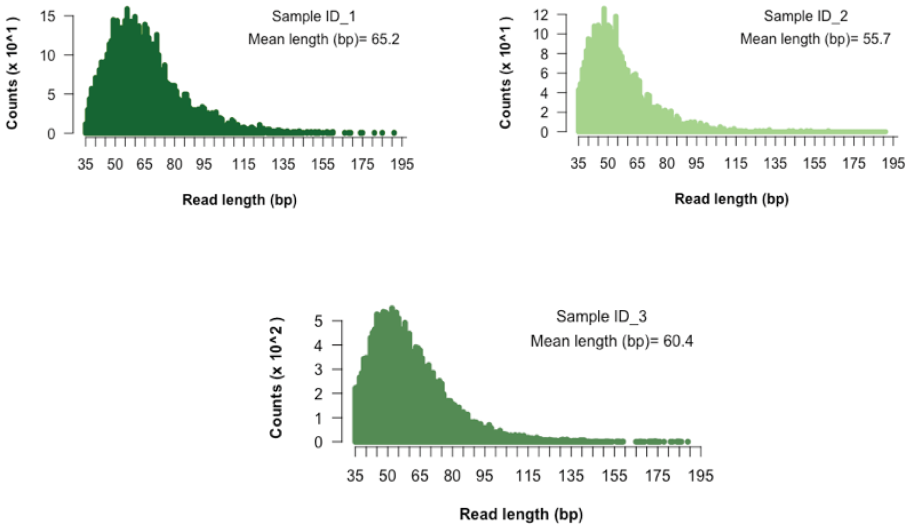

b

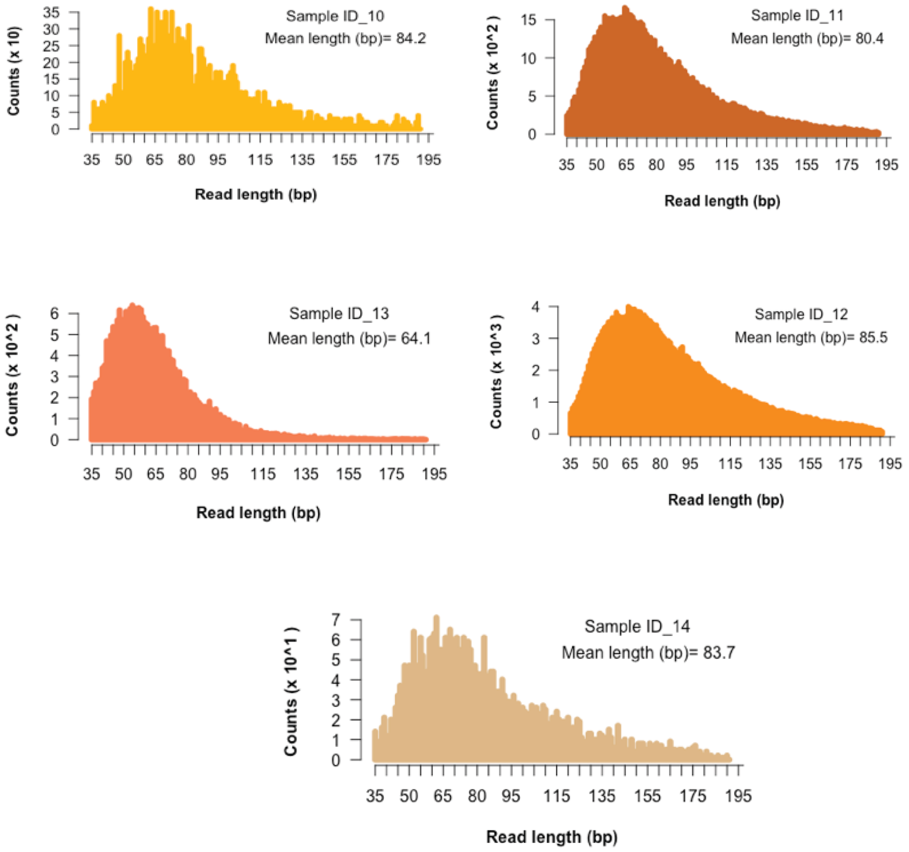

Supplementary Figure 3. DNA fragmentation profiles for ancient dental samples. A 5’ -1 enrichment of the purine guanine is observed for all mitogenomes. (**a**) sample 1, (**b**) sample 2, (**c**) sample 3, (**d**) sample 10, (**e**) sample 11, (**f**) sample 12, (**g**) sample 13, (**h**) sample 14. DNA fragmentation profiles were generated using MapDamage v.2.

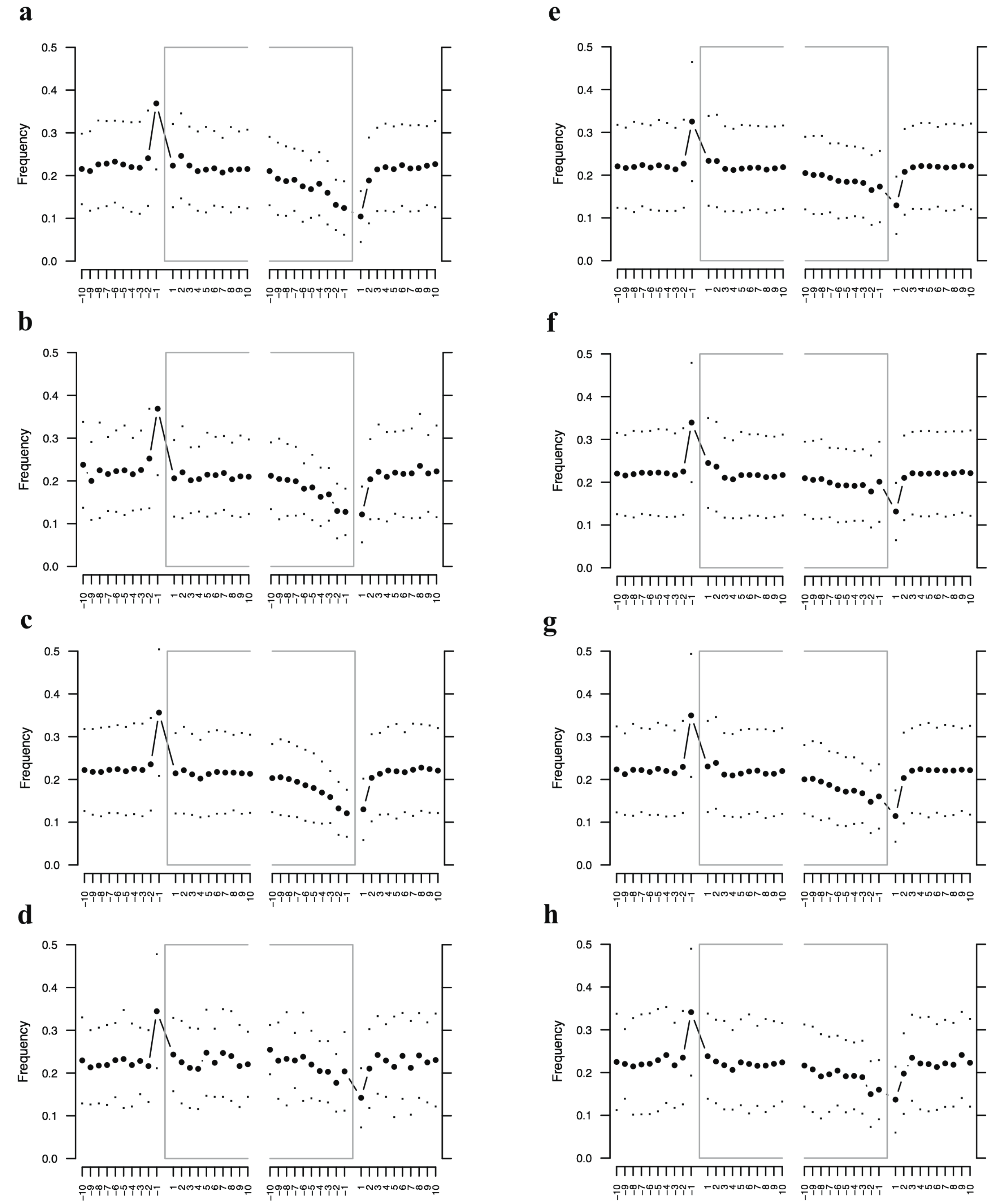

Supplementary Table 1. Mitochondrial haplogroup frequencies of selected indigenous American populations.

Study		Haplogroup frequency (%)					References[b]
	N[a]	A	B	C	D	X	
AMER. SOUTHWEST							
Ancestral Puebloan	178	11	65	14	10	‡	3, 4, 30, 31
Ancestral Southwest[d]	21	9.5	81	9.5	0	‡	13
Zuni	50	16	76	8	0	‡	7, 10, 13, 15, 16
Akimel O'odham	146	5	47	48	1	‡	7, 10, 13, 15, 16, 27
Jemez	71	0	86	4	0	10	7, 10, 13, 15, 16, 25
Fremont	32	0	75	13	6	0	32
MESOAMERICA							
Huichol	102	25.5	27.5	47.3	0	0	39
Purepecha	65	83.1	1.5	10.8	4.6	0	39
Ancient Nahua	80	57.5	25	6.3	11.2	0	5, 8, 33, 34
Nahua	590	57	29	8	6	0	7, 10, 13, 15, 16, 21, 38
Ancient Otomi	10	30	30	0	40	0	33
Otomi	275	50	17	25	8	‡	21, 37, 38
Tepehua	53	64	26.7	5.5	3.8	0	38
Mazateco	41	53.7	34.1	2.4	9.8	0	39
Colonial Mixtec	41	54	24	17	5	0	29
Mixtec	123	67	23	7	3	0	7, 10, 13, 21, 28
Zapotec	100	41	24	30	5	0	7, 10, 13, 28
Mixe	68	38	29	24	9	0	7, 10, 13, 28
Ancient Teotihuacan (Teopancazco)	29	55	21	17	7	0	40
Ancient Maya (Xcaret)	24	88	4	8	0	‡	6
Ancient Maya (Copán)[c]	9	0	0	8	1	‡	17
Maya	447	54.5	24	13.5	8	0	2, 21, 25, 26, 39
CENTRAL AMERICA							
Chibcha	539	52	46	0	2	0	35, 36, 41, 42, 43, 44
Chorotega	30	73	10	0	17	0	41
CARIBBEAN							11
Ancient Taino	24	0	0	75	25	‡	12
Ancient Ciboney	15	7	0	60	33	0	11
CHOCOAN							
Emberá	65	18.5	52.3	26.2	3	0	45, 46
Wounan	31	29	19	48	3	0	45
ANDEAN							
Ancient Andean[e]	187	24.5	39	24.5	12	‡	9, 14, 19, 20, 22, 23
Quechua	51	20	61	8	12	0	1, 18
Aymara	205	5	72	11	12	0	1, 18

Notes:

‡Testing for haplogroup X was not performed, and the haplogroup assignment of at least one individual from this population is given as "other" (i.e., not A, B, C, or D).

[a]Excludes individuals with haplogroups indicative of European or African admixture, as well as individuals for whom haplogroup assignment was not determined.

[b]Pooled published data from: 1=Bert et al. 2001; 2=Boles et al. 1995; 3=Carlyle et al. 2000; 4=Carlyle 2005; 5=De la Cruz et al. 2008; 6=Gonzalez-Oliver et al. 2001; 7=Kemp 2006; 8=Kemp et al. 2005; 9=Kemp et al. 2009; 10=Kemp et al. 2010; 11=Lalueza fox et al. 2001; 12=Lalueza Fox et al. 2003; 13=LeBlanc et al. 2007; 14=Lewis et al. 2007; 15=Lorenz and Smith 1996; 16=Malhi et al. 2003; 17=Merriwether et al. 1997; 18=Merriwether et al. 1995; 19=Moraga et al. 2001; 20=Moraga et al. 2005; 21=Peñaloza-Espinosa et al. 2007; 22=Rothhammer et al. 2009; 23=Schurr et al. 1990; 24=Shinoda et al. 2006; 25=Smith et al. 1999; 26=Torroni et al. 1992; 27=Torroni et al. 1993; 28=Torroni et al. 1994; 29= Warinner et al. 2012; 30= Snow et al. 2010; 31= Snow et al. 2011; 32= Parr et al., 1996; 33= Mata-Míguez et al. 2012; 34= Solórzano, 2006; 35= Melton et al. 2007; 36= Baldi and Crawford 2016; 37= Sandoval et al. 2009; 38= Gorostiza et al. 2012; 39= Gonzales Martín et al. 2015; 40=Alvarez-Sandoval et al. 2015; 41= Melton et al. 2013; 42= Santos et al. 1994; 43 = Kolman et al. 1995; 44= Batista et al. 1995; 45= Kolman and Bermingham 1997; 46= Usme-Romero et al. 2013.

[c]The unusual haplogroup distribution found in this study is likely an artifact of poor preservation and amplification bias.

[d]Ancient DNA in this study was recovered from cultural artifacts rather than human remains.

[e]Ancient DNA haplogroup frequency data from Sicán and Sipán (Shimada et al. 2005; Shimada et al. 2004) were excluded because individuals within the tomb assemblages exhibited close maternal relationships.

Supplementary Table 2. Quantification results and volume of extract added to the library preparation. A maximum of 100 ng DNA was added per library, and volumes added were adjusted to 30 ul in each reaction.

Sample ID	Library ID	ng/ul	ul for 100 ng	DNA extract added (ul)
1	J31-127	0.79	126.6	30
2	1-133CE1	13.10	7.6	7.6
3	J64	0.59	168.4	30
4	J22-33	2.28	43.9	30
5	J24-96	1.84	54.3	30
6	J39-154	6.40	15.6	15.6
7[c]	J130C	12.80	7.8	7.8
7[d]	J24-130	1.68	59.5	30
8	C3-4	3.82	26.2	26.2
9	C58-26	0.72	139.3	30
10	P14-1AC	0.69	143.7	30
11	P20-13A	0.78	127.6	30
12	P2-16A	0.21	476.2	30
13	P25-6A	0.42	238.1	30
14	P16	0.40	250.0	30

Supplementary Table 3. Descriptive statistics of Jícaro, Paquimé, and Convento sequence data. See associated spreadsheet file.

Supplementary Table 4. Extended haplotype for each mitogenome.

Sample ID	H	Range	Haplotype extended description
**Jícaro**			
1	B2d	5-1321; 1334-16567	41T 73G 263G 315.1C 498d 499A 750G 827G 1438G 2706G 3547G 4122G 4123G 4769G 4820A 4977C 6473T 7028T 8281-8289d 8860G 8875C 9682C 9950C 11177T 11719A 13590A 14766T 15326G 15535T 16092C 16183C 16189C 16217C 16519C
2	B2d	11-1336; 1381-1515; 1517-1577;1583-1590; 1592-1600; 1602-1948; 1954-2674; 2682-2779; 2781-3105; 3107-3580; 3582-8152; 8172-16464; 16480-16555	73G 263G 315.1C 498d 499A 750G 827G 1438G 1698T 2706G 3547G 4122G 4123G 4769G 4820A 4977C 6285A 6473T 7028T 8173T 8182T 8269A 8281-8289d 8860G 8875C 9767T 9682C 9950C 11177T 11719A 13590A 14766T 15326G 15535T 15523N 16183C 16189C 16217C 16519C
3	B2d	1-16567	73G 152C 263G 315.1C 498d 499A 750G 827G 1438G 2706G 3547G 4122G 4123G 4769G 4820A 4977C 6473T 7028T 8281-8289d 8860G 8875C 9682C 9950C 11177T 11719A 13590A 14766T 15326G 15535T 16182C 16183C 16189C 16217C 16519C
**Paquimé and Convento**			
10	C1c5	3-187; 225-627; 629-986; 988;1037; 1039; 1043-1801; 1834-1839; 1842-1846; 1848-2083; 2125-2310; 2312-2314; 2316-2593; 2595-2905; 2907; 2939-3364; 3366-4249; 4285-5496; 5498; 5502; 5504-6675; 6677-6690; 6692-6734; 6736-6768; 6770-6777; 6787; 6789-6797; 6799-7044; 7046-7272; 7274-7312; 7315-7433; 7435-7440; 7442-7548; 7550-7554; 7556-7750; 7752-8073; 8075-8350; 8427-8471; 8473-8475; 8477-8709; 8711-1092; 1094-10322; 10464-10806; 10808-11135; 11137-11782; 11784-11944; 11949-11960; 11962-12315; 12333-12721; 12723-12876; 12878-13389; 13391-14179; 14190-14521; 14655-14751; 14753-15834; 15898; 15900-15952; 15954-15969; 15971-16150; 16152-16490	73G 249d 263G 290d 291d 489C 750G 1438G 1888A 2706G 3552A 4715G 4769G 7028T 7196A 8584A 8701G 8860G 9540C 9545G 10527T 10873C 11617C 11719A 11914A 12705A 12888T 13263G 14318C 14662G 14664T 14671T 14766T 14783C 15043A 15301A 15326G 15487T 15930A 16223T 16298C 16325C 16327T
11	C1c1a	1-16569	73G 215G 249d 263G 290d 291d 315.1C 489C 750G 1438G 1888A 2706G 3552A 4715G 4769G 7028T 7196A 8584A 8701G 8860G 9540C 9545G 10398G 10400T 10873C 11719A 11914A 12705T 12978G 13263G 14318C 14766T 14783C 15043A 15301A 15326G 15487T 15930A 16223T 16298C 16325C 16327T
12	B2f	1-16569	73G 263G 315.1C 499A 709A 750G 827G 1438G 2706G 3547G 3796G 3996T 4769G 4820A 4977C 5277C 6473T 7028T 8281-8289d 8860G 9950C 10535C 11177T 11719A 13590A 13833G 14766T 15326G 15535T 16183C 16189C 16217C 16519C
13	C1c1a	1-16566	73G 215G 249d 263G 290d 291d 489C 750G 1438G 1888A 1899A 2706G 3552A 4715G 4769G 7028T 7196A 8584A 8701G 8860G 9540C 9545G 10398G 10400T 10873C 11719A 11914A 12705T 12978G 13263G 14318C 14766T 14783C 15043A 15301A 15326G 15487T 15803A 15930A 16223T 16298C 16325C 16327T
14	B2a	5-1617; 1619-1922; 1924-16543; 16545-16561; 16563	73G 263G 499A 750G 827G 1438G 2706G 3547G 4769G 4820A 4977C 6335T 6473T 7028T 8281-8289d 8860G 9950C 11177T 11719A 13158G 13590A 14766T 15326G 15535T 16111T 16183C 16189C 16217C 16261T 16287T 16390A 16483A 16519C

References Cited

Álvarez-Sandoval BA, Manzanilla LR, González-Ruiz M, Malgosa A, Montiel R. (2015). Genetic Evidence Supports the Multiethnic Character of Teopancazco, a Neighborhood Center of Teotihuacan, Mexico (AD 200-600). *PLoS ONE* 10(7): e0132371.

Baldi NF, Crawford MH. (2016). Population History and Mitochondrial Genetic Substructure of the Rama Amerindians from Nicaragua. *Human Biology* 88(2):168-181.

Batista O, Kolman CJ, Bermingham E. (1995). Mitochondrial DNA diver- sity in the Kuna Amerinds of Panama. *Human Molecular Genetics* 4:921–929.

Bert, F, Corella, A, Gene M, Perez-Perez, A, Turbon, D. (2001). Major mitochondrial DNA haplotype heterogeneity in highland and lowland Amerindian populations from Boliva. *Human Biology* 73:1-16.

Boles, TC, Snow, CC, Stover, E. (1995). Forensic DNA testing on skeletal remains from mass graves: a pilot project in Guatemala. *Journal of Forensic Sciences* 40:349-355.

Carlyle, SW, Parr, Ryan L, Hayes, M Geoffrey, O'Rourke, Dennis H. (2000). Context of maternal lineages in the Greater Southwest. *American Journal of Physical Anthropology* 113:85-101.

Carlyle, S. (2005). Discerning the origins of the Anasazi with mtDNA haplogroups. In D.M. Reed (Ed), *Biomolecular archaeology: genetic approaches to the past* (pp. 93-127). Occasional Paper 32. Center for Archaeological Investigations, Southern Illinois University, Carbondale.

De la Cruz I, Gonzalez-Oliver A, Kemp BM, Roman JA, Smith DG, Torre-Blanco A. (2008). Sex identification of children sacrificed to the ancient Aztec rain gods in Tlateloco. *Current Anthropology* 49(3):519-526.

González-Martín A, Gorostiza A, Regalado-Liu L, Arroyo-Peña S, Tirado S, Nuño-Arana I, Rubi-Castellanos R, Sandoval K, Coble MD, Rangel-Villalobos H. (2015). Demographic History of Indigenous Populations in Mesoamerica Based on mtDNA Sequence Data. *Plos One* 10 (8): e0131791.

González-Oliver, A, Márquez-Morfín, L, Jiménez, JC, Torre-Blanco A. (2001). Founding Amerindian mitochondrial DNA linages in ancient Maya from Xcaret, Quintana Roo. *American Journal of Physical Anthropology* 116:230-235.

Gorostiza A, Acunha-Alonzo V, Regalado-Liu L, Tirado S, Granados J, Samano D, Rangel-Villalobos H, Gonzalez-Martin A. (2012). Reconstructing the History of Mesoamerican Populations through the Study of the Mitochondrial DNA Control Region. *PLoS ONE* 7(9): e44666.

Kolman CJ, Bermingham E. (1997). Mitochondrial and nuclear DNA diversity in the Choco and Chibcha Amerinds of Panama. *Genetics* 147:1289– 1302.

Kolman CJ, Bermingham E, Cooke R, Ward RH, Arias TD, Guionneau- Sinclair F. (1995). Reduced mtDNA diversity in the Ngöbe Amerinds of Panama. *Genetics* 140:275–283.

Kemp BM, Reséndez A, Román Berrelleze JA, Malhi RS and Smigh DG. (2005). An analysis of ancient Aztec mtDNA from Tlatelolco. In *Biomolecular archaeology: genetic approaches*

to the past. Reed DM, ed. Occasional Paper 32. Center for Archaeological Investigations, Southern Illinois University, Carbondale. Pp. 32-46.

Kemp, BM. (2006). *Mesoamerica and Southwest prehistory, and the entrance of humans into the Americas: mitochondrial DNA evidence* (Unpublished doctoral dissertation). University of California, Davis, CA.

Kemp B, González-Oliver A, Malhi R, Monroe C, Schroeder K, McDonough J, Rhett G Resendéz A, Peñalosa-Espinosa R, Buentello-Malo L, Gorodesky C, Smith DG. (2010). Evaluating the farming/language dispersal hypothesis with genetic variation exhibited by populations in the Southwest and Mesoamerica. *Proceedings of the National Academy of Science* 107(15):6759-6764.

Kemp B, Tung T, Summar M. (2009). Genetic continuity after the collapse of the Wari Empire: mitochondrial DNA profiles from Wari and post-Wari populations in the ancient Andes. *American Journal of Physical Anthropology* 140:90-91.

Lalueza Fox C, Gilbert MTP, Martínez Fuentes AJ, Calafell F, Bertranpetit J. (2003). Mitochondrial DNA from Pre-Columbian Ciboneys from Cuba and the prehistoric colonization of the Caribbean. *American Journal of Physical Anthropology* 121:97-108.

Lalueza Fox C, Luna Calderón F, Calafell F, Morera B, Bertranpetit J. (2001). MtDNA from extinct Tainos and the peopling of the Caribbean. *Annals of Human Genetics* 65: 137-151.

LeBlanc SA, Cobb LS, Kemp BM, Smiley FE, Carlyle SW, Dhody AN, Benjamin T. (2007). Quids and Aprons: Ancient DNA from Artifacts from the American Southwest. *Journal of Field Archaeology* 32(2): 161-175.

Lewis CM, Buikstra J, Stone A. (2007). Ancient DNA and genetic continuity in the south central Andes. *Latin American Antiquity* 18(2):145-160

Lorenz, Joseph G, Smith, David Glenn. (1996). Distribution of four founding mtDNA haplogroups among native North Americans. *American Journal of Physical Anthropology* 101: 307-323.

Malhi RS, Mortensen HM, Eshleman JA, Kemp BM, Lorenz JG, Kaestle FA, Johnson JR, Gorodezky C, Smith DG. (2003). Native American mtDNA prehistory in the American Southwest. *American Journal of Physical Anthropology* 120:108-124.

Mata-Míguez J, Overholtzer L, Rodríguez-Alegría E, Kemp BM, Bolnick DA. (2012). The genetic impact of aztec imperialism: Ancient mitochondrial DNA evidence from Xaltocan, Mexico. *American Journal of Physical Anthropology* 149:504–516.

Melton PE, Briceño I, Gómez A, Devor E, Bernal J, Crawford M. (2007). Biological relationship between central and South American Chibchan speaking populations: Evidence from mtDNA. *American Journal of Physical Anthropology* 133:753–770.

Melton PE, Baldi NF, Barrantes R, Crawford MH. (2013). Microevolution, migration, and the population structure of five Amerindian populations from Nicaragua and Costa Rica. *American Journal of Human Biology* 25:480–490.

Merriwether DA, Reed DM and Ferrell RE. (1997). Ancient and contemporary mitochondrial DNA variation in the Maya. In *Bones of the Maya: studies of ancient skeletons*. Whittington SL and Reed DM, eds. Washington, DC: Smithsonian Institution Press.

Merriwether, DA, Rothhammer, F, Ferrell, RE. (1995). Distribution of the four founding lineage haplotypes in Native Americans suggests a single wave of migration for the New World. American *Journal of Physical Anthropology* 98:411-430.

Moraga M, Aspillaga E, Santoro C, Standen V, Carvallo P, Rothhammer F. (2001). Análisis de ADN mitocondrial en momias del norte de Chile avala hipótesis de origen amazónico de poblaciones andinas. *Revista Chilena de Historia Natural* 74:719-726.

Moraga, Mauricio, Santoro Calogero, Standen, Vivien, Carvallo, Pilar, Rothhammer, Francisco. (2005). Microevolution in prehistoric Andean populations: chronologic mtDNA variation in the desert valleys of Northern Chile. *American Journal of Physical Anthropology* 127:170-181.

Parr RL, Carlyle SW, O'Rourke DH. (1996). Ancient DNA analysis of Fremont Amerindians of the Great Salt Lake Wetlands. *American Journal of Physical Anthropology* 507–518.

Peñaloza-Espinosa, RI, Arenas-Aranda, D, Cerda-Flores, RM, Buentello-Malo, L, González-Valencia, G, Torres, J, Alvarez, B, Mendoza, I, Flores, M, Sandoval, L, Loeza, F, Ramos, I, Muñoz, L, Salamanca, F.(2007). Characterization of mtDNA haplogroups in 14 Mexican indigenous populations. *Human Biology* 79(3):313-320.

Rothhammer F, Santoro C, Poulin E, Arriaza B, Moraga M, Standen V. (2009). Archaeological and mtDNA evidence for tropical lowland migrations during the Late Archaic/Formative in Northern Chile. *Revista Chilena de Historia Natural* 82:543-552.

Sandoval K, Buentello-Malo L, Peñaloza-Espinosa R, Avelino H, Salas A, Calafell F, Comas D. (2009). Linguistic and maternal genetic diversity are not correlated in Native Mexicans. *Human Genetics* 126:521–531.

Santos MR, Ward H, Barrantes R. (1994). mtDNA variation in the Chibcha Amerindian Huetar from Costa Rica. *Human Biology* 66: 963-977.

Schurr, TG, Ballinger, SW, Gan, YY, Hodge, JA, Merriwether, DA, et al. (1990). Amerindian mitochondrial DNAs have rare Asian mutations at high frequencies, suggesting they derived from four primary maternal lineages. *American Journal of Human Genetics* 46:613–623.

Shinoda K, Adachi N, Guillen S, Shimada I. (2006). Mitochoondrial DNA analysis of ancient Peruvian highlanders. *American Journal of Physical Anthropology* 131(1):98-107.

Smith, DG, Malhi, RS, Eshleman, J, Lorenz, JG, Kaestle, FA. (1999). Distribution of mtDNA haplogroup X among Native North Americans. *American Journal of Physical Anthropology* 110:271-284.

Snow M, Durand KR, Glenn Smith D. (2010). Ancestrasl Puebloan mtDNA in context of the greater southwest. *Journal of Archaeological Science* 37: 1635-1645.

Snow M, Shafer H, Glenn Smith D. (2011). The relationship of the Mimbres to other southwestern and Mexican populations. *Journal of Archaeological Science* 38: 3122-3133.

Solórzano E. 2006. *De la Mesoamerica prehispánica a la colonial: la huella del DNA antiguo.* (Unpublished doctoral dissertation). Universidad Autónoma de Barcelona: Barcelona.

Torroni A, Chen Y-S, Semino O, Santachiara-Beneceretti AS, Scott CR, Lott MT, Winter M, Wallace, DC. (1994). MtDNA and Y-chromosome polymorphisms in four Native American populations from Southern Mexico. *American Journal of Human Genetics* 54: 303-318.

Torroni, A, Schurr, TG, Cabell, MF, Brown, MD, Neel, JV, Larsen, M, Smith, DG, Vullo, CM, Wallace, DC. (1993). Asian affinities and continental radiation of the four founding Native American mtDNAs. *American Journal of Human Genetics* 53(3): 563–590.

Torroni, Antonio, Schurr, Theodore G, Yang, Chi-Chuan, Szathmary, Emöke JE, Williams, Robert C, Schanfield, Moses S, Troup, Gary A, Knowler, William C, Lawrence, Dale N, Weiss, Kenneth M, Wallace, Douglas. (1992). Native American mitochondrial DNA analysis indicates that the Amerind and the Nadene populations were founded by two independent migrations. *Genetics* 130:153–162.

Usme-Romero S, Alonso M, Hernandez-Cuervo H, Yunis EJ, Yunis JJ. (2013). Genetic differences between Chibcha and Non-Chibcha speaking tribes based on mitochondrial DNA (mtDNA) haplogroups from 21 Amerindian tribes from Colombia. *Genetics and Molecular Biology* 36:149–157.

Warinner C, García NR, Spores R, Tuross N. (2012). Disease, Demography, and Diet in Early Colonial New Spain: Investigation of a Sixteenth-Century Mixtec Cemetery at Teposcolula Yucundaa. *Latin American Antiquity* 23:467–489.
